# Congenital malformation of fetus in a pregnancy following spontaneous ovulation in a case of premature ovarian failure

**DOI:** 10.4103/0974-1208.63121

**Published:** 2010

**Authors:** Priya Selvaraj, Kamala Selvaraj, Megha Agrawal, Gunjan Singh

**Affiliations:** Department of Reproductive Medicine, GG Hospital, Fertility Research Centre, Chennai, Tamilnadu, India

**Keywords:** Congenital malformation, donor oocyte program, genetic screening, karyotyping, premature ovarian failure, spontaneous ovulation

## Abstract

Premature ovarian failure (POF), that is, amenorrhea before 40 years of age can be attributed to a variety of etiologies. Approximately 1% of women before 30 years are diagnosed with POF. Spontaneous ovulation leading to pregnancy in POF is even a rarer entity. We report a case where congenital malformations were diagnosed in a fetus following spontaneous ovulation in a case of POF. A 33-year-old woman presented to our center with primary infertility. On complete work up, she was diagnosed with POF and conceived with hormone replacement therapy and donor oocyte program. She delivered a healthy female baby through caesarean section. The patient reviewed later with amenorrhea of 40 days and pregnancy was confirmed. However, during antenatal follow-up congenital anomalies in fetus were diagnosed sonographically. The decision for termination of pregnancy was taken. To conclude, we recommend large-scale retrospective analysis that would define medical guidelines in cases of pregnancy following spontaneous ovulation in POF.

## INTRODUCTION

Premature or secondary ovarian failure also known as hypergonadotropic hypogonadism is defined as amenorrhea occurring prior to 40 years of age.[1] It may occur from a variety of etiologies. The diagnosis may remain elusive despite a thorough diagnostic work-up, including a karyotype, specific antibody studies, or an ovarian biopsy. Approximately 1% of women over the age of 30 are estimated to have premature ovarian failure (POF).[2] Anecdotal reports of pregnancies despite apparent ovarian failure have appeared. Although some apparently occurred without any therapy, most cases involved women who were on some form of estrogen replacement.[3] We describe one case report from our center that throws light on the rare possibility of spontaneous ovulation in POF and its outcomes.

## CASE REPORT

**Figure 1:**
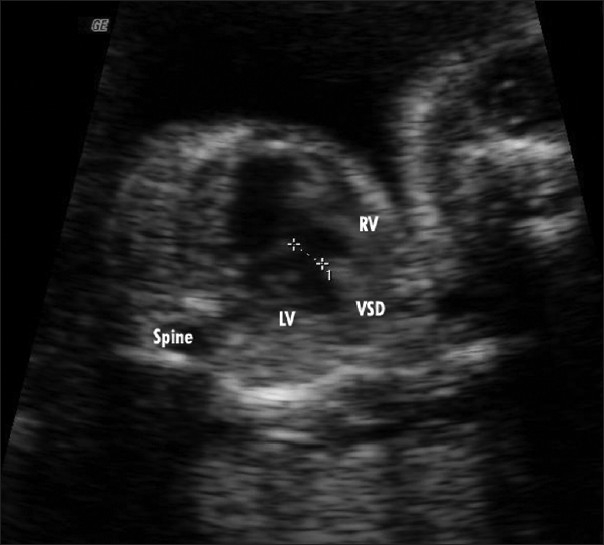
Ventricular septal defect

A 33-year-old lady married for 9 years visited our center for primary infertility. She was hypomenorrhic and menstruating only on progesterone withdrawal since 4 years. Transvaginal ultrasonography revealed uterine size of 7.0 × 3.1 cm. Both ovaries were not visible [Figure 1]. Hormonal assay on third day of periods showed FSH of 41.74 mIU/ml and LH of 26.33 mIU/ml. Her prolactin and thyroid profile were within normal limits. Husband's semen analysis was normal. Karyotype analysis of wife was normal. A diagnostic laparoscopy showed normal uterus size, right tubal block, patent left tube, and small corrugated ovaries on both sides. A diagnosis of POF was made based on all the above findings. The couple was counseled for donor oocyte program, while the patient herself was advised for hormone replacement therapy (HRT) in view of her previous menstrual history. Patient was ready for HRT but wanted more time for deciding about the donor oocyte program. During this time period, she took a second opinion at a different center where wedge biopsy of both ovaries was performed, which further confirmed the diagnosis of POF.

She came back to our center for further treatment after a gap of 2 years. With due consent of the couple, assisted reproductive technology (ART) program was carried out using donor oocytes and she conceived in the third attempt of embryo transfer (donor age 29 and 30 years, six eggs, of which two fertilized, were transferred (1 of G-II-III and 1 of G-II)). Her antenatal period was uneventful. She delivered a healthy female baby weighing 2.49 kg by caesarean section in April 2007. Subsequently, the patient came to us after 1 year for follow-up during which she was lactating and not menstruating. An ultrasonography at this follow-up revealed a uterus size of 4.9 × 2.7 cm. Ovaries were not visible. Patient was once again advised HRT but she preferred to defer treatment for a couple of months. During this time, she had a spontaneous period after which she came to us with 40 days amenorrhea.

**Figure 2:**
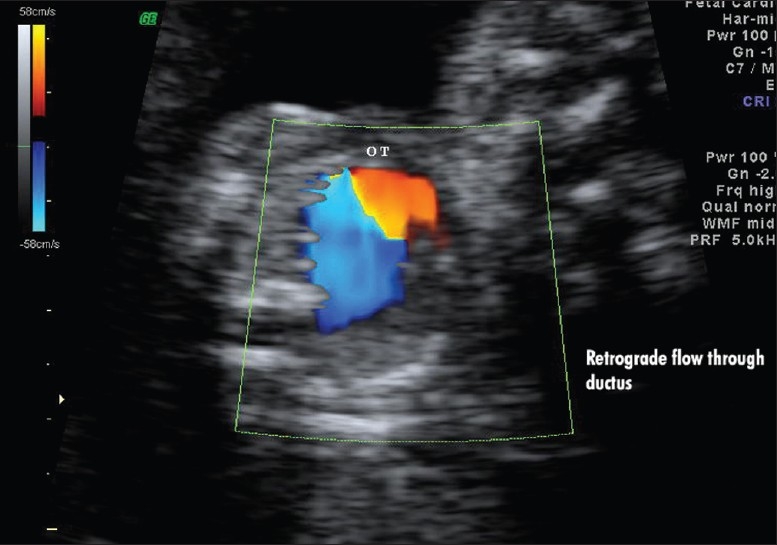
Cardiac anomaly

**Figure 3:**
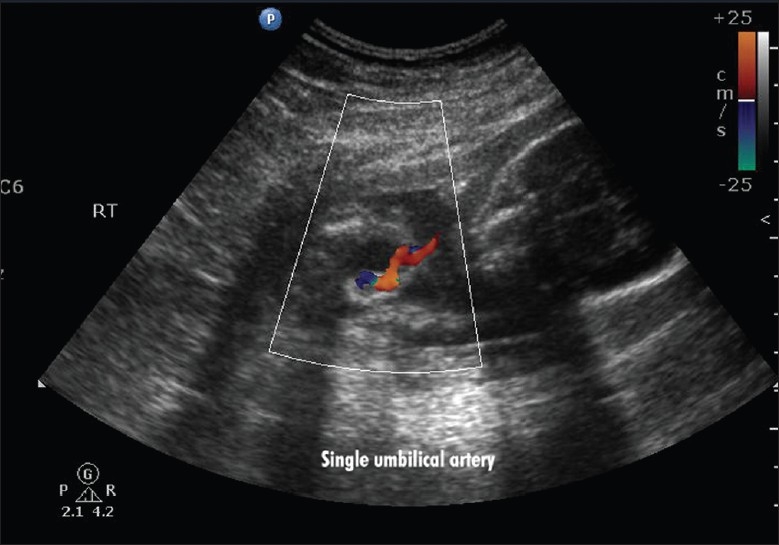
Single umbilical artery

**Figure 4:**
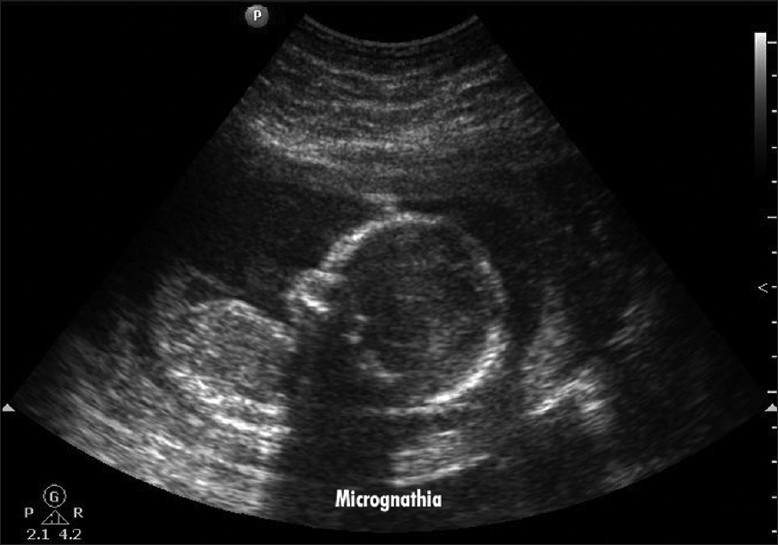
Micrognathia

**Figure 5:**
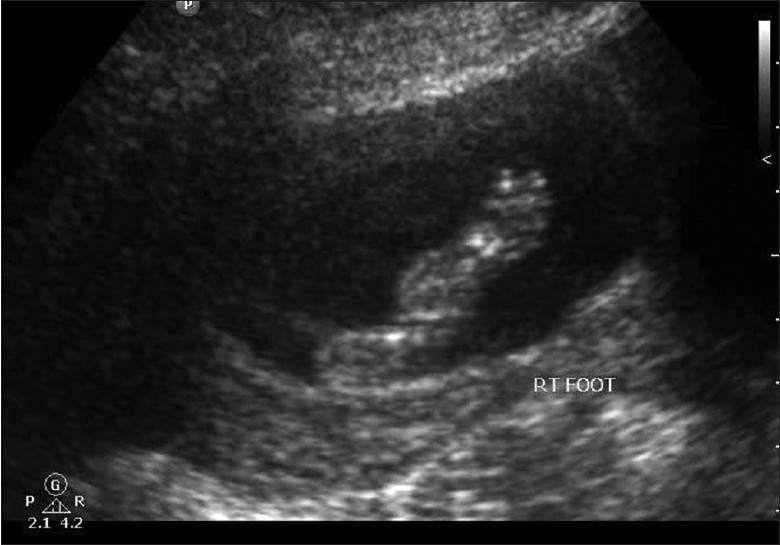
Prominent heel

**Figure 6:**
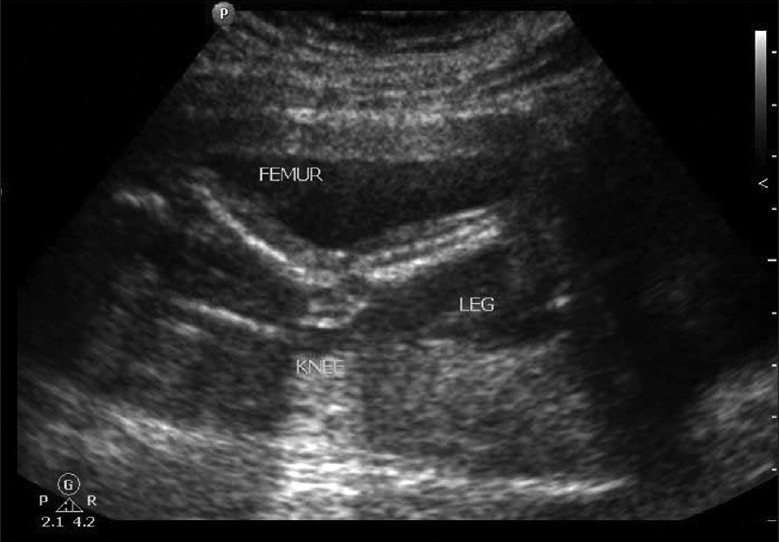
Persistent extended lower limbs

**Figure 7:**
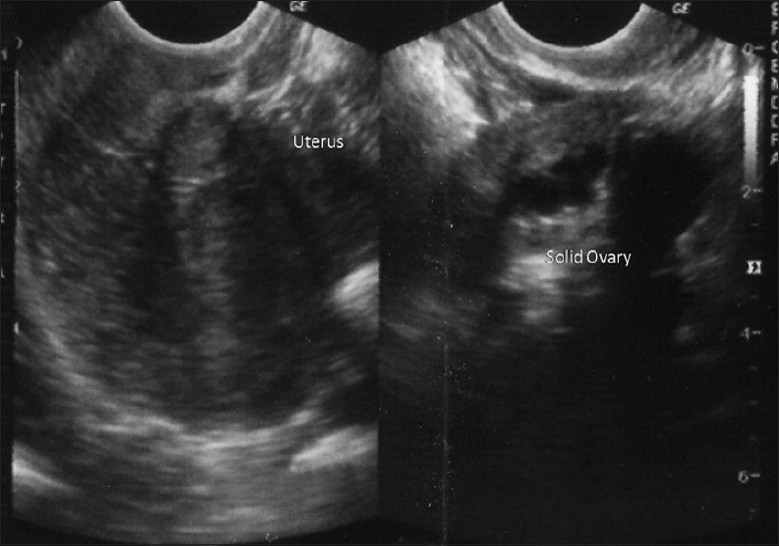
Pelvic USG

Scan showed an intrauterine gestational sac corresponding to 5 weeks and 5 days. It was indeed a case of natural conception following a spontaneous ovulation, which is a rare occurrence although not unknown in cases of POF. Patient was on regular antenatal follow-up. A first trimester screening was normal, but a level II scan for fetal anomalies revealed single umbilical artery, micrognathia, dysmorphic face, multiple cardiac anomalies (large ventricular septal defect (VSD), pulmonary atresia, double outlet right ventricle, narrow branch pulmonary artery), and persistently extended lower limb, partially flexed upper limb [Figures 2–7]. In view of these findings, medical termination of pregnancy (MTP) was advised. MTP was attempted with misoprostol in conventional dosage schedule of 200 μg per vaginally every 3-4 hours for three doses, but owing to failure of induction and nonprogress, patient underwent a hysterotomy. The anomalous female fetus of 400 gm was delivered. The karyotype of the fetus was done, which revealed normal chromosomes with no other chromosomal anomalies. Postoperative period was uneventful.

## DISCUSSION

Once periods have ceased for 6 months and the diagnosis of POF is secure, a few women experience a return to fertility and achieve pregnancy. Spontaneous pregnancy has been reported in rare instances.[4,5] The findings that make the possibility of spontaneous pregnancy more likely are fluctuating FSH measurements, the ability to identify ovaries on ultrasound, and the association with autoimmunity or chemotherapy.[6] While normal menopause is generally an irreversible condition, POF is characterized by intermittent ovarian function in half of these young women, with intermittent estrogen production and even ovulation despite the presence of high gonadotropin levels. Some women with spontaneous POF have been found to possess apparently normal primordial follicles.[7] Spontaneous conception is possible with a lifetime chance of about 10-15%. The mechanism might involve the possibility of elevated gonadotropin causing downregulation of gonadotropin receptors and restoration of the sensitivity of the few remaining ovarian follicles by lowering of serum gonadotropin with estrogen therapy.[8]

It is recommended that patients with POF should be treated by a trial HRT and should have close monitoring for ovulation prior to resorting to oocyte donation. It is also to be noted that since majority of them are subjected to initial HRT to promote uterine growth to optimum size, the use of abortifacients invariably fails owing to alteration in both numbers and responsiveness of target receptors. These women thus end up in surgical interventions like hysterotomy.

Regarding our case, the probable mechanism for the return of fertility might be the fluctuating hormone levels. The possibility of congenital anomalies in this fetus may be a result of the poor quality of oocytes because of the altered FSH and LH levels and small uterus size. We recommend all such pregnancies to undergo a thorough antenatal genetic screening and level-II USG to rule out congenital anomalies. We thus feel the need for a larger retrospective study, which might help in knowing the incidence of such a correlation and defining the management guidelines in such cases.
